# Prognosis and risk factors of patients with upper urinary tract urothelial carcinoma and postoperative recurrence of bladder cancer in central China

**DOI:** 10.1186/s12894-019-0457-5

**Published:** 2019-04-18

**Authors:** Qingwei Wang, Tao Zhang, Junwei Wu, Jianguo Wen, Deshang Tao, Tingxiang Wan, Wen Zhu

**Affiliations:** 1 Department of Urology, The First Affiliated Hospital of Zhengzhou University, Zhengzhou, 450052 Henan China; 2 Department of Pediatric Surgery, The First Affiliated Hospital of Xinxiang Medical University, Xinxiang, 453100 Henan China

**Keywords:** Upper urinary tract urothelial carcinoma, Survival analysis, Bladder cancer recurrence, Prognostic factor, Risk factor

## Abstract

**Background:**

To investigate the prognostic risk factors and postoperative recurrence of bladder cancer in patients with upper urinary tract urothelial carcinomas (UTUCs).

**Methods:**

Data of 439 UTUC patients were retrospectively analyzed. Follow-up and analysis of smoking effects, consumption of traditional Chinese medicine containing aristolochic acid, history of bladder cancer, age, sex, presence or absence of diabetes mellitus (DM), metformin use, tumor characteristics (number, location, stage, grade), and open or laparoscopic surgery on the prognosis of UTUCs were performed. Cox proportional hazard regression analysis was performed to analyze the relationship between various factors and the postoperative survival rate. The survival rate was analyzed using the Kaplan-Meier method. Moreover, logistic regression analysis was performed to analyze the relationship between the above mentioned factors and postoperative recurrence of bladder cancer.

**Results:**

Overall, 439 patients met, including 236 males (53.7%) and 203 females (46.3%), the criteria for the final statistical analysis, and the average age was 66.7 years. The 1-, 3-, and 5-year overall survival rates of 439 UTUC patients were 90.0, 76.4, and 67.7%, respectively. The 5-year survival rates of T1, T2, T3, and T4 patients were 90.2%, 78%, 43.8%, and 18.5%, respectively. Factors influencing the long-term survival rate of UTUC patients were smoking, taking traditional Chinese medicine containing aristolochic acid, history of bladder cancer, age, tumor size, tumor stage, tumor grade, and lymph node metastasis. The risk factors related to postoperative bladder cancer recurrence were advanced tumor stage, high grade tumor, preoperative ureteroscopy, ureteral urothelial carcinoma, no postoperative bladder perfusion chemotherapy and DM without metformin use.

**Conclusions:**

Advanced tumor stage and presence of a high-grade tumor were risk factors for not only poor UTUC prognosis but also BC recurrence. In addition, preoperative ureteroscopy, ureteral urothelial carcinoma and DM without metformin use were high risk factors for BC recurrence, whereas regular postoperative bladder perfusion chemotherapy was a protective factor.

## Background

Upper urinary tract urothelial carcinoma (UTUC) is a relatively rare tumor, accounting for approximately 10% of all renal tumors and only 5% of all urothelial carcinomas [1, 2], with an estimated annual incidence of almost two cases per 100,000 inhabitants in Western countries. The epidemiology and disease presentation of UTUCs in the Chinese population, however, are quite different from those in Western populations. First, UTUCs account for 20–30% of all transitional cell carcinomas (TCCs) in China, which is more common than that in Western countries [3, 4]. Second, UTUCs are more common in women than in men, with a ratio of 1.3:1 [4]. Third, the incidence of renal pelvis urothelial carcinoma is twice more among Chinese populations than among Western populations. TCC of the ureter accounts for more than half of UTUCs [5, 6] and about 25% of all renal carcinomas in China [3]. Finally, contrary to findings in Western countries, UTUC with an advanced stage, large tumor size, and lymph node metastasis (LNM) are less likely to progress in female Chinese patients than in male Chinese patients [7]. This may be associated with diverse environmental factors, such as pure-arsenic exposure in drinking water and consumption of aristolochic acid in Chinese herbs [3, 8].

However, the epidemiology and biological characteristics of UTUCs in the Chinese population have not gained research focus, and data are limited. Moreover, it remains unclear whether the following factors are related to prognosis and postoperative recurrence of bladder cancer (BC) in UTUC patients: diabetes mellitus (DM), metformin use in DM patients, preoperative ureteroscopy, and location of UTUC. Currently, radical nephroureterectomy (RNU) is the gold standard for treating UTUCs [9]. The incidence of intravesical tumor recurrence after operation is 20–50% [10]. Notably, 44% of patients with BC recurrence have invasive disease (≥pT1 stage), and 82–89% of BC recurrences occur within 2 years after RNU [11]. Hence, identifying high risk factors of BC recurrence after RNU is a major concern for UTUC patients.

Although a few studies have assessed predictive or risk factors of UTUCs and/or BC recurrence in Chinese people [3, 12], data were mainly obtained from the population of the North and Southeast of China, not from the central areas of China. Since the epidemiology and disease characteristics may be related to different areas, along with factors influencing survival and tumor recurrence remain controversial, we developed a database of UTUC in our region to investigate the prognostic risk factors of UTUC and BC recurrence in central China. We hope that our research will contribute to UTUC treatment options and reduce the risk of recurrence of BC after RNU.

## Methods

### Subjects

Data of patients with upper urinary tract tumors (UUTTs) from the Department of Urology of our hospital (the largest hospital in China, located in central China) were collected from March 2011 to February 2017. The inclusion criteria were as follows: 1. patients with unilateral UTUCs; 2. those who underwent RNU; 3. those with postoperative pathological confirmation of UTUC; and 4. those with complete clinical data and follow-up. The exclusion criteria were as follows: 1. patients with bilateral UTUC; 2. those with a history of UTUC awaiting renal transplantation; 3. those who received conservative treatment or simple nephrectomy; and 4. those with incomplete or missing clinical data.

Finally, 481 patients with UUTTs were selected. Among them, 12 patients did not undergo RNU, 8 had incomplete clinical data, 11 were lost to follow-up, 5 had squamous cell carcinoma, 4 had adenocarcinoma, and 2 were diagnosed with sarcoma. Finally, 439 patients with UTUC were included in this study. All patients underwent RNU with bladder cuff resection, and each patient was confirmed as having UTUC based on pathological reports.

### Data records

The collected clinical data of UTUC patients included smoking status, history of taking Chinese herbs containing aristolochic acid, history of BC, DM, and DM with/without metformin use, age, sex, tumor characteristics (number, location, stage, grade, type), operation method, preoperative ureteroscopy, postoperative bladder perfusion chemotherapy, and BC recurrence (Table 1).

**Table 1:** The related prognostic influencing factors of UTUCs and the risk factors of bladder cancer recurrence in this study

Influential factors	Classification	No. of patients (%)
Sex	Male	236(53.7%)
	Female	203(46.3%)
Age	<50 yr	132(30.1%)
	≥50 yr	307(69.9%)
Smoking	Yes	204(46.5%)
	No	235(53.5%)
TCHAA	Yes	173(39.4%)
	No	166(60.6%)
History of BC	Yes	95(9.2%)
	No	344(90.8%)
DM	Yes	133(30.3%)
	No	306(69.7%)
DM + metformin use	Yes	53(39.8%)
	No	80(60.2%)
Number of tumors	Single	278(63.3%)
	Multiple	161(36.7%)
Tumor location	Renal pelvis	253(57.6%)
	Ureter	140(42.4%)
Tumor stage	T1-T2	246(56.0%)
	T3-T4	193(44.0%)
Tumor size	<3 cm ≥3 cm	255(58.1%) 184(41.9%)
Tumor grade	G1-G2	352(80.2%)
	G3	87(19.8%)
LNM	Yes No	86(19.6%) 353(80.4%)
Preoperative Ureteroscopy	Yes	139(31.7%)
	No	300(68.3%)
Operation mode	Laparoscopic surgery	312(71.1%)
	Open surgery	127 (28.9%)
BC recurrence		89(20.3%)
	Renal pelvis carcinoma	30(33.7%)
	Ureteral carcinoma	59(66.3%)
	242 cases of PBPC	28(11.6%)
	197 cases of no PBPC	61(31.0%)
	DM	63(70.9%)
	DM with Metformin use	15(16.9%)
	DM without metformin use	48(53.9%)

*THCAA* taking Chinese herbs containing aristolochic acid, *LNM* lymph node metastasis, *PBPC* postoperative bladder perfusion chemotherapy, *DM* diabetes mellitus, *BC* bladder cancer

The criteria for identifying the influencing factors were as follows: a smoking index (number of cigarettes per day×years of smoking) above 200 was considered smoking. A history of taking Chinese medicine containing aristolochic acid was considered if at least 1 of the following criteria were met: 1) taking Chinese herbal medicine or Chinese patent medicine containing caulis aristolochiae manshuriensis; the duration of ingestion included the continuous use of caulis aristolochiae manshuriensis for > 15 days or the discontinuous use for > 2 months and 2) the continuous or intermittent use of other types of Chinese herbal or proprietary medicines containing aristolochic acid ingredients for > 6 months. Those aged > 50 years were considered as advanced in age. A low tumor location indicated ureteral urothelial carcinoma (UUC). The tumor clinical staging was performed according to the American Joint Committee on Cancer (AJCC) 2002; the tumor grading was assessed based on the WHO pathological grading system of malignant urothelial cancer 2004.

### Follow-up

All patients were followed up regularly by telephone or internet contact, which was recorded in the electronic medical records (EMR) system of our hospital. Full-time urologists were responsible for following up patients according to the UTUC guidelines, and the patients were urged to undergo their medical examinations on time. All examination results during the follow-up period were recorded in detail. Postoperatively, cystoscopy was performed every 3 months within 2 years to assess for recurrence, then once every 6 months in the 3rd–4th year, and then once a year thereafter. During the follow-up time, other routine examinations, including blood routine tests, liver and kidney function tests, abdominal B-scan ultrasonography, chest X-ray every 6 months, and abdominal computed tomography (CT) once a year, were performed. Based on the treatment guidelines for UTUCs and patients’ wishes, bladder perfusion chemotherapy following RNU for patients with UTUC was not a compulsory treatment. Depending on whether bladder instillation was performed after the operation, patients were divided into two groups: the perfusion group and the non-perfusion group. Perfusion was performed once a week for a maximum of 8 weeks and then once a month for 1 year following the operation. All data were recorded and monitored using the Jiahe EMR System. The survival rate, BC recurrence rate, and risk factors of BC recurrence were analyzed. The mortality and cause of death in the follow-up period were assessed based on data obtained from the family members, and the remaining data were obtained from the EMR system or the attending doctor.

### Statistical analysis

Each influencing clinical factor was quantified (Table 1). A Cox proportional hazard regression model was used to evaluate the relationship between the risk factors and postoperative survival rate. Survival time was defined as the difference between follow-up time and operative time in months. The Kaplan-Meier method was used to analyze the survival rate. Furthermore, factors affecting postoperative BC recurrence were analyzed using a logistic regression model. SPSS 20.0 software was used to analyze the data, and a *P* value < 0.05 was considered statistically significant.

## Results

Overall, 439 patients were included in the final statistical analysis. Among the 439 patients, 236 were males (53.7%) and 203 were females (46.3%), and the average age was 66.7 years. The follow-up period ranged from 18 to 84 months, with an average of 62.5 months. Factors included in the analysis are shown in Table 1. Tumor-associated death, BC recurrence rate, survival rate, risk factors for UTUCs, and BC recurrence were analyzed to predict the prognosis of patients with UTUC.

### Survival rate

**Fig. 1:**
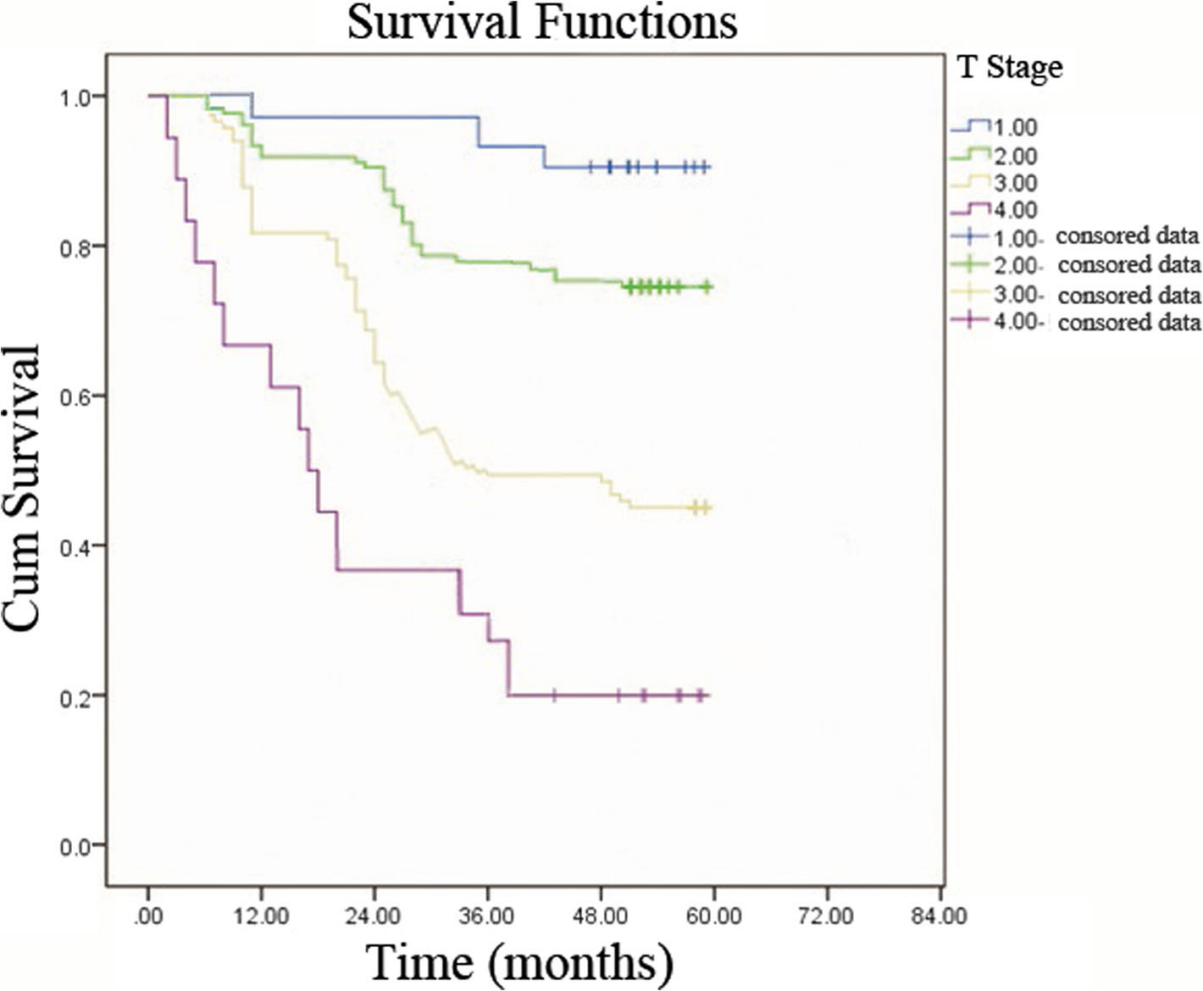
The survival curve of the UTUC patients used by Kaplan-Meier method

The 1-, 3-, and 5-year overall survival rates of the 439 patients were 90.0, 76.4, and 67.7%, respectively. The 5-year survival rates of patients with T1, T2, T3, and T4 were 90.2, 78, 43.8, and 18.5%, respectively (Fig. 1).

### Effects of cox regression analysis on the prognosis of UTUCs

Cox regression analysis was performed to eliminate confounding factors. Eight factors, namely smoking, consumption of Chinese medicine containing aristolochic acid, history of BC, age, tumor stage, tumor grade, tumor size, and LNM, had a significant effect on survival and were identified as prognostic factors (*P* < 0.05). This suggested that smoking, taking Chinese medicine containing aristolochic acid, history of BC, older age, advanced tumor stage, high-grade tumors, larger tumors, and presence of LNM were risk factors of UTUC patients with poor prognosis. Sex, number of tumors, tumor location, DM, and mode of operation had no effect on the prognosis (*P* > 0.05). The results of the analysis of factors influencing survival time and prognosis are shown in Table 2.

**Table 2:** The outcomes of multiple COX regression analysis in the prognosis of UTUC

Variable	B	SE	Wald	Exp(B)	P
Smoking	0.964	0.214	20.207	2.381	< 0.01
THCAA	0.633	0.214	6.870	1.883	0.009
Age	0.357	0.169	4.468	0.700	0.035
Sex	0.002	0.145	0.000	1.002	0.991
Number of tumors	0.061	0.159	0.149	1.063	0.700
History of BC	0.698	0.228	9.356	2.010	0.002
LNM	1.994	0.353	31.915	7.343	< 0.01
Tumor size	0.449	0.158	8.086	0.638	< 0.01
Tumor location	0.162	0.260	0.390	0.850	0.532
Tumor stage	1.304	0.157	69.163	3.683	< 0.01
Tumor grade	2.157	0.298	52.291	8.646	< 0.01
Operation mode	0.472	0.378	1.559	1.603	0.212
DM	0.742	0.357	3.458	2.204	0.076

*THCAA* taking Chinese herbs containing aristolochic acid, *LNM* lymph node metastasis, *DM* diabetes mellitus

### Analysis of risk factors of postoperative BC recurrence

Among the 439 patients, 89 (20.3%) had BC recurrence, and their data were analyzed using the binary logistic regression model. We found that advanced tumor stage, presence of a high-grade tumor, preoperative ureteroscopy, DM without metformin use, and UUC were risk factors of postoperative BC recurrence. Patients with 1 or more than 1 of the risk factors were more likely to experience BC recurrence. Furthermore, regular postoperative bladder perfusion chemotherapy was a protective factor for recurrent BC. The results of the analysis of high risk factors for postoperative BC recurrence are shown in Table 3.

**Table 3:** The results of multiple logistic regression analysis of recurrent BC after RNU in UTUC patients

Variable	B	SE	Wald	Exp(B)	P
Age	0.170	0.525	0.105	1.186	0.746
Operation mode Number of tumors	0.016 0.030	0.322 0.389	0.002 0.006	1.016 1.030	0.960 0.939
LNM	0.228	0.574	0.158	1.256	0.691
Advanced tumor stage	1.064	0.300	12.548	2.899	< 0.01
Tumor location (UUC)	2.835	0.627	20.440	16.949	< 0.01
High-grade tumor	2.576	0.590	19.048	13.140	< 0.01
PBPC	−1.065	0.299	5.564	2.025	0.018
Preoperative Ureteroscopy	1.010	0.290	7.920	11.292	0.003
DM without Metfoemin use	2.156	0.354	6.785	3.872	0.032

*LNM* lymph node metastasis, *PBPC* postoperative bladder perfusion chemotherapy, *RUN* radical nephroureterectomy, *UUC* ureteral urothelial carcinoma, *DM* diabetes mellitus

## Discussion

In this study, we found that smoking, consuming Chinese medicine containing aristolochic acid, history of BC, old age, advanced tumor stage, presence of high-grade tumor, larger tumor size, and LNM were predictive factors for worse survival of UTUC. In addition, tumor stage, tumor grade, preoperative ureteroscopy, UUC, and DM without metformin use were risk factors of BC recurrence, whereas regular postoperative bladder perfusion chemotherapy and DM with metformin use were protective factors for BC relapse. Because the accurate prediction of the prognosis of UTUC patients could contribute to risk stratification and devising therapeutic options for urologists or oncologists, we analyzed each predictor in detail.

The incidence of UTUCs has been increasing each year, and 60% of UTUCs are found to have already developed into invasive tumors at diagnosis compared with 15–25% of bladder tumors [1, 13]. UTUCs have a peak incidence in individuals aged 70–90 years and are three times more common in men in Western countries [1, 14]; these findings differed from those of our study in which the male-to-female ratio was 1:1.16. Previous studies showed that the survival rate of UTUC patients was related to the tumor stage and grade [4]. A multifactorial analysis of 252 UTUC cases by Hall et al. [15] revealed that tumor stage was the only indicator of postoperative survival. A study of 434 patients with UTUCs by Munoz and Ellison [16] showed that the 5-year survival rate of patients with Tis tumors was 95.1% and that of patients with local tumors was 88.9%; however, the 5-year survival rate of patients with distant metastasis was only 16.5%. In our study, tumor stage and grade were risk factors influencing the survival of postoperative UTUC patients. A significant difference in 5-year survival rate occurred between low tumor staging (T1–T2) and high tumor staging (T3–T4), as well as between different tumor grades (G1–G2 and G3–G4). This suggests that early diagnosis and treatment with periodic follow-up are crucial for improving the survival rate.

UTUCs are prone to relapse and have a recurrence rate of 16–58% after surgery; thus, the current standard treatment of UTUCs is nephroureterectomy with bladder cuff resection [16]. The study also showed that the surgical approach (laparoscopic or open surgery) is not an influencing factor of postoperative survival. Mufti [17] also proposed that the surgical method (laparoscopy or open surgery) was not a prognostic factor, which is consistent with our results.

For most people, age is a risk factor of UTUC [18]. Raman et al. [19] investigated 13,800 patients with upper urinary tract tumors and showed that mortality owing to UTUCs increased with age. Similarly, we showed that compared with patients aged < 50 years, the relative risk of postoperative mortality increased among patients aged > 50 years. It is hypothesized that as the patient’s age increases, the biological behavior of the tumor changes, with a decline in immune system function. LNM can be used to predict prognosis, especially for patients without lymph node dissection. Multicenter studies confirmed that LNM is closely related to higher tumor invasiveness, such as higher stage, higher grade, and distant metastasis. LNM can independently affect tumor recurrence and survival rate; hence, it is an independent risk factor for UTUC prognosis [20]. Our study also suggested that LNM was significantly associated with poor prognosis, which is consistent with the result of previous research. Tumor size has been also confirmed as a prognostic factor in some malignant tumors. The results of Simone’s study [21] on UTUCs revealed that metastasis-free survival was closely related with tumor size: when the tumor diameter was < 3 cm, there was no metastasis within 5 years, whereas when it was > 3 cm, the 5-year metastasis-free survival rate was 67%. Our study also revealed that tumor size was a risk factor of UTUC, wherein a larger tumor diameter indicated worse prognosis.

It is known that smoking is a prognostic factor of UTUC. A study [22] revealed that smoking is the main risk factor of UTUC and lower urinary tract urothelial cancer. The incidence of urothelial carcinoma in smokers is three times greater than that in nonsmokers, probably owing to a mutation in tumor protein p53, chromosomal changes, immune regulation, etc. Our study also confirmed that smoking was a risk factor of poor prognosis in UTUC patients. Moreover, we found that consumption of herbal medicines such as Longdanxiegan pills, Paishi granule, Paishi decoction, and caulis aristolochiae manshuriensis, which contain aristolochic acid, also affected the prognosis of UTUC patients. DNA compounds can be formed in vivo under the influence of aristolochic acid [23], leading to the A-T base-pair proto-oncogene mutation, activation of RAS, and dysfunction of the cancer suppressor gene P53. Thus, smoking may play an important role in the occurrence of UTUCs. Additionally, our study confirmed that BC was a prognostic factor for UTUC, which is consistent with the findings of Nuhn [24]. This may be due to the similar pathogenesis of UTUC and BC or a missed diagnosis of UTUC in some BC patients (especially for ureteral TCCs). For the latter reason, urologists who examine patients with hematuria may often be satisfied with a BC diagnosis and omit ureteral TCCs that could also lead to BC. This explains our finding that a prior history of muscle-invasive urothelial carcinoma of the bladder was significantly associated with an increased risk of disease recurrence and cancer-specific death in UTUC patients. Therefore, further adjuvant treatment, ureteroscopy, CT urography, and close follow-up should be performed after BC surgery.

It is generally believed that UTUC patients should be treated with radical RNU with the resection of bladder cuff resection [25]. However, the recurrence rate of BC is very high even after radical surgery. The incidence of BC relapse after operation in patients with TCC of the urinary tract is 30–70% [26], while the rate in our study was slightly lower at 20.3%. In the case of postoperative BC recurrence, a tumor located in the ureter, especially at the lower end of the duct, is considered a high-risk factor for BC recurrence. Zigeuner [27] suggested that a ureteral tumor is prone to spread into the bladder because of its close anatomical position. This could be due to the mechanical stress caused by a higher urinary flow rate and larger chamber pressure, facilitating tumor cell metastasis. Furthermore, in recent years, studies worldwide have found that the risk of BC relapse in ureteral carcinoma is significantly higher than that of a renal pelvis carcinoma after RNU [28], which corresponds to the findings of our study. This, in some degree, supports the cancer cell implantation theory as the mechanism of BC recurrence. Fang et al. [28] analyzed the risk factors of BC recurrence after primary UTUC radical resection in 438 cases and found that high-grade ureteral cancer and multiple tumors were high-risk factors of BC recurrence. Therefore, a close follow-up with cystoscopy is necessary for patients with a high-grade, high-stage tumor and ureteral cancer (especially those with lower urinary tract ureteral cancer). These results indicate that tumor size and LNM are not independent risk factors of BC recurrence in UTUC patients after operation. However, there is limited research on whether tumor size and LNM affect bladder tumor recurrence; thus, more samples and multicenter studies are required.

Marchioni et al. [29] confirmed that preoperative ureteroscopy increases the risk of postoperative BC recurrence, which is consistent with the results our study. If the cancer cell implantation theory as the main mechanism of BC recurrence is accepted, there is greater risk of cancer cell exfoliation and implantation in the bladder after ureteroscopy examination. Thus, preoperative ureteroscopy should not be the primary and routine method for diagnosing UTUCs if imaging diagnosis is relatively clear; rather, a safer imaging method should be performed.

Because UTUC is closely related to BC and is associated with a higher BC recurrence rate, postoperative intravesical instillation chemotherapy has been widely used to prevent BC recurrence in postoperative UTUC patients. Many studies showed that postoperative bladder perfusion chemotherapy can effectively reduce the recurrence rate of BC after UTUC surgery [30]. Our study showed that postoperative bladder perfusion chemotherapy was a protective factor of BC recurrence after RNU. Therefore, UTUC patients could benefit from early diagnosis and regular postoperative intravesical instillation chemotherapy.

Recent studies have demonstrated that the incidence and mortality of tumor patients with DM were significantly higher than those of patients without DM. In this study, we found that UTUC patients with DM were more likely to experience BC recurrence. Among postoperative UTUC patients, the BC recurrence rate in those with DM was approximately four times that in patients without DM, and the result was significantly different. However, the specific mechanism remains unknown, which could be closely associated with DM itself and the associated hyperglycemia, hyperinsulinemia, and lipid metabolism disorder. In a study of 251 people with non-muscle-invasive bladder carcinoma, DM was an independent risk factor for disease recurrence [31]. Liu’s in vitro experiments showed that high doses of insulin can promote the proliferation of urinary tract epithelial cells [32]. DM is often accompanied by insulin resistance and hyperinsulinemia; thus, Liu’s study results may be a plausible explanation for easily recrudescent BC after surgery in UTUC patients with DM. Metformin is currently a first-line oral hypoglycemic drug for the treatment of type 2 DM. Currently, research has shown that metformin can reduce the risk of cancer by inhibiting the proliferation of tumor cells in vivo and in vitro [33, 34]. Our study found that after RNU, the BC recurrence rate in UTUC patients with DM who did not take metformin was significantly higher than that in UTUC patients with DM who took metformin. Our study supported the fact that metformin can reduce the recurrence rate of BC in patients with UTUC and DM, which has not been reported thus far. Metformin mainly activates the AMPK pathway, promotes the expression of the P53 gene, and inhibits the mTOR pathway, thereby inhibiting tumor cell proliferation and reducing insulin and insulin-like growth factor levels [35]. However, whether these mechanisms also apply to the occurrence and progression of UTUCs in DM patients remain under study.

This study has some limitations. First, the research data represent a retrospective review of findings at a single center. Second, patients who were not surgically treated were not included in the analysis. Finally, lack of information on molecular biomarkers may reduce the strength of the findings. Therefore, further studies are necessary to confirm the role of molecular biomarkers as predictors for worse pathological outcomes of UTUCs.

## Conclusions

In this study, the 5-year survival rate of UTUC patients from central China was not high compared with that of UTUC patients from other regions. We found that advanced tumor stage and presence of a high-grade tumor were risk factors for not only poor UTUC prognosis but also BC recurrence. In addition, preoperative ureteroscopy and DM without metformin use were high risk factors for BC recurrence, whereas postoperative bladder perfusion chemotherapy was a protective factor. Therefore, we suggest that UTUC patients with the above risk factors for BC recurrence should compulsorily undergo regular bladder perfusion chemotherapy. In addition, we suggest that preoperative ureteroscopy should not be exercised except when preoperative imaging diagnosis is difficult.

## Abbreviations

BC: bladder cancer
CT: computed tomography
DM: diabetes mellitus
EMR: electronic medical records
LNM: lymph node metastasis
RNU: radical nephroureterectomy
TCC: transitional cell carcinomas
TCCs: transitional cell carcinomas
UTUC: upper urinary tract urothelial carcinoma
UTUCs: upper urinary tract urothelial carcinomas
UUC: ureteral urothelial carcinoma
UUTT: upper urinary tract tumor
